# Chitosan-Modified Coconut Shell Activated Carbon for Efficient Hexavalent Chromium Removal from Aqueous Solution

**DOI:** 10.3390/polym18101237

**Published:** 2026-05-19

**Authors:** Danyun Lei, Weiyi She, Xiaoyu Chen, Lei You, Ying Zheng, Byoung-Suhk Kim

**Affiliations:** 1 College of Urban Construction, Wuchang Shouyi University, Wuhan 430064, China; 2 Department of Organic Materials & Textile Engineering, Jeonbuk National University, Jeonju-si 561-756, Jeollabuk-do, Republic of Korea; 3 Department of BIN Convergence Technology, Jeonbuk National University, Jeonju-si 561-756, Jeollabuk-do, Republic of Korea

**Keywords:** chitosan, coconut shell activated carbon, chemical modification, hexavalent chromium, adsorption

## Abstract

Chitosan (CS) was employed to modify coconut shell activated carbon (CAC) to fabricate a composite adsorbent for wastewater treatment. By integrating the functional groups of CS with the high specific surface area of CAC through chemical modification, the resulting CS-AC composite exhibited significantly enhanced adsorption performance toward hexavalent chromium (Cr(VI)) in aqueous solutions. The effects of key parameters, including adsorbent dosage, initial Cr(VI) concentration, contact time, temperature, and solution pH on the adsorption efficiency were systematically investigated. Under optimal conditions, the CS-AC composite achieved a Cr(VI) removal efficiency of up to 99.04%. Kinetic and isotherm modeling revealed that the adsorption process followed the pseudo-second-order kinetic model and was well described by the Langmuir isotherm. Regeneration studies conducted over five consecutive adsorption-desorption cycles demonstrated that the composite retained a high removal efficiency of 98.10%, indicating excellent reusability. These findings suggest that the CS-AC composite is a promising and effective adsorbent for the removal of Cr(VI) from contaminated water.

## 1. Introduction

With the rapid advancement of global industry, heavy metal pollution in water has become increasingly severe. Heavy metal-containing wastewater poses a serious threat to human health and has emerged as a global environmental concern [1,2,3]. Among various heavy metals, hexavalent chromium (Cr(VI)) is considered one of the most toxic pollutants due to its acute toxicity, mutagenicity and carcinogenicity [4,5,6]. Chromium-containing wastewater is mainly generated from industries such as electroplating, chemical processing, and metallurgy, with the electroplating industry being the largest contributor [7,8,9,10]. The hazards associated with chromium-containing wastewater depend on the oxidation state of chromium. In aqueous systems, chromium exists primarily as Cr(III) and Cr(VI). Compared to Cr(III), Cr(VI) exhibits significantly higher toxicity, stronger carcinogenic and mutagenic effects, and greater environmental mobility due to its potent oxidizing nature, posing severe risks to both ecological systems and human health [11,12]. Motivated by these concerns, considerable research efforts have been directed toward the treatment of heavy metal-contaminated wastewater.

Adsorption is an important method for removing Cr(VI) from wastewater [13,14,15]. Commercial activated carbon adsorption has been widely applied for the treatment of Cr(VI)-containing wastewater due to its simple operation, environmental friendliness, and reliability [15]. Nevertheless, the relatively limited functional groups and uneven pore distribution of activated carbon restrict its applications in wastewater treatment. Various modification strategies, including chemical, physical and biological approaches, have been explored to enhance its adsorption efficiency. Physical modification encompasses high-temperature heat treatment, microwave radiation, and ultrasonic modification [16,17]. Chemical modification includes surface oxidation, reduction, acid-base treatment, and loading modification [18,19]. Surface oxidation modification involves treating activated carbon with common oxidants (e.g., HClO, Cl_2_, HNO_3_, H_2_O_2_, KMnO_4_, O_3_), which adjusts the population of oxygen-containing surface groups, thereby improving its hydrophilicity and surface acidity [20,21]. Surface reduction modification introduces a reducing agent at elevated temperatures to increase the number of basic groups on the activated carbon surface, enhancing its surface alkalinity, non-polarity and hydrophobicity [19]. Acid-base modification employs non-oxidizing and non-reducing acid or base solutions to remove surface impurities and modify surface chemistry, ultimately improving adsorption performance [18]. Loading modification involves immobilizing metal elements onto the activated carbon surface, enhancing adsorption capacity and efficiency through complexation between the loaded species and target adsorbates [22]. Biological modification refers to the functionalization of activated carbon by immobilizing microorganisms, enzymes, or biological molecules (e.g., proteins, polysaccharides) onto its surface. This approach aims to enhance adsorption performance or introduce specific selectivity toward target pollutants. By combining the physical adsorption advantages of activated carbon with the specificity of biological materials, this strategy has become prevalent in wastewater treatment [23]. Modifying activated carbon surfaces with biomolecules such as DNA, proteins, and chitosan alters the surface chemical properties, thereby improving chelation capacity for heavy metal ions.

Chitosan (CS) is a natural polysaccharide derived from deacetylation of chitin, with a molecular structure composed of D-glucosamine and N-acetyl-D-glucosamine units linked by β-(1,4)-glycosidic bonds [24]. As an environmentally friendly biomaterial, CS exhibits excellent biocompatibility, biodegradability, antibacterial properties, and low immunogenicity. CS is also recognized for its low cost and non-toxic nature [25,26]. The high removal efficiency of CS for heavy metal ions is primarily attributed to its polycationic functional groups, such as hydroxyl and amino groups, which enable specific chelation with target metal ions [27]. However, the practical application of CS in wastewater treatment is hindered by several inherent drawbacks, including excessive solubility in acidic media, a pronounced swelling tendency, low specific surface area, and poor mechanical strength [28]. To address these limitations, various strategies, such as composite modification and cross-linking, have been employed to enhance its mechanical stability, reduce hydrophilicity, and improve performance under acidic conditions.

By introducing chitosan onto activated carbon, this modification strategy retains the excellent physical adsorption properties of activated carbon while imparting the unique chemical chelation capability of CS. The resulting synergistic effect, arising from the high specific surface area of activated carbon and the specialized functional groups of CS, significantly enhances the adsorption efficiency for heavy metal ions. This approach offers a promising direction for the development of efficient and environmentally friendly adsorbents for heavy metal removal.

In this work, chitosan was immobilized onto the surface of activated carbon to construct a composite adsorbent via a simple modification strategy. The aim was to integrate the high specific surface area and well-developed pore structure of activated carbon with the abundant amino and hydroxyl functional groups of chitosan. It is hypothesized that this synergistic combination can enhance Cr(VI) adsorption through multiple mechanisms, including electrostatic attraction and surface complexation. Compared with pristine activated carbon and chitosan, the introduction of chitosan is expected to significantly improve the adsorption capacity and affinity toward Cr(VI), particularly at low pH. Therefore, this work provides a feasible and efficient approach for developing environmentally friendly adsorbents for the removal of heavy metal ions from aqueous systems.

## 2. Materials and Methods

### 2.1. Materials

Chitosan (CS) (degree of deacetylation ≥ 95.0%) and acetic acid (purity ≥ 99.5%) were purchased from Shanghai Aladdin Biochemical Technology Co., Ltd. (Shanghai, China) and National Pharmaceutical Group Chemical Reagents Co., Ltd., China (Shanghai, China), respectively. Coconut shell activated carbon was obtained from Suzhou Tanxuan Windmill Activated Carbon Co., Ltd., Suzhou, China. Sodium hydroxide, the chromium standard solution (1000 μg·mL^−1^), and ethylenediaminetetraacetic acid disodium salt (EDTA-2Na, purity ≥ 99.0%) were supplied by National Pharmaceutical Group Chemical Reagents Co., Ltd., the National Center for Analysis and Testing of Nonferrous Metals and Electronic Materials (Beijing, China), and Tianjin Hengxing Chemical Reagents Manufacturing Co., Ltd., Tianjin, China, respectively. The (1 + 1) phosphoric acid solution and the hexavalent chromium color developer (containing diphenylcarbazide, ethanol, and (1 + 9) sulfuric acid) were prepared in the laboratory. All chemicals used in this work were of analytical grade and employed without further purification.

### 2.2. The Modification of Activated Carbon

CS was first dissolved in a 2% (*v*/*v*) acetic acid solution under constant stirring until complete dissolution, yielding a homogeneous and transparent solution. According to a CS to activated carbon mass ratio of 1:6, a predetermined amount of CAC powder was weighed and slowly added to the chitosan solution. The mixture was continuously stirred to ensure uniform dispersion of the CAC. Following this, the mixture was left to stand for 24 h to allow for complete impregnation of chitosan onto the activated carbon surface. The impregnated solid was then collected by filtration and subsequently soaked in a 1 mol·L^−1^ NaOH solution for 24 h to promote chitosan curing. The resulting product was thoroughly rinsed with deionized water until the filtrate reached a neutral pH, thereby removing any residual alkali and acetic acid. Finally, the washed sample was dried in a vacuum oven at 60 °C for 24 h, then ground and sieved to obtain a uniform modified activated carbon powder, which was stored in a desiccator for future use. The resulting modified activated carbon was denoted as CS-AC.

### 2.3. Characterizations

The morphologies of CS-AC adsorbents were analyzed by scanning electron microscope (S4800, Hitachi Limited, Tokyo, Japan). The specific surface area of the adsorbents was determined using a surface area and pore size analyzer (ASAP 2460, Micromeritics, Norcross, GA, USA) via nitrogen adsorption and the Brunauer–Emmett–Teller (BET) method. Fourier-transform infrared spectroscopy (FTIR, Nicolet 6700, Thermo Fisher Scientific, Waltham, MA, USA) was employed to examine the interactions between the adsorbent and chromium species.

### 2.4. Calculation of Cr(VI) Concentration

The concentration of Cr(VI) was determined spectrophotometrically at 540 nm after complexation with 1,5-diphenylcarbazide under acidic conditions. A calibration curve was established using K_2_Cr_2_O_7_ standard solutions in the concentration range of 0 to 1 mg·L^−1^, which showed excellent linearity (R^2^ = 0.998). The Cr(VI) removal efficiency was calculated according to the following Equation (1):

(1) $$\eta \;=\;\frac{c_{0}-c_{e}}{c_{0}}\;\times \;100\%$$

where c_0_ and c_e_ are the initial and equilibrium concentrations of Cr(VI) (mg·L^−1^), respectively. All experiments were conducted in triplicate, and the results are presented as the mean values ± standard deviation (SD).

### 2.5. Adsorption and Desorption Experiments

All Cr(VI) adsorption experiments were conducted in batch mode. Preliminary experiments were performed using 25 mL of synthetic wastewater containing 70 mg·L^−1^ of Cr(VI) at pH 1.0 and 25 °C, with oscillating for 2 h. To investigate the effects of key parameters on adsorption efficiency, single-factor experiments were carried out by varying one parameter while keeping the others constant. The parameters studied included adsorbent dosage (10–100 mg), solution pH (1.0–10.0), initial Cr(VI) concentration (20–80 mg·L^−1^), temperature (25–55 °C at 10 °C intervals), and contact time (30–180 min). Each experiment was performed in triplicate, and the data are presented as mean ± standard deviation.

Adsorption-desorption experiments were performed to evaluate the reusability of CS-AC. Each adsorption cycle involved contacting the adsorbent with 25 mL of 70 mg·L^−1^ Cr(VI) solution under optimal conditions for 2 h. The Cr(VI)-loaded adsorbent was then collected by centrifugation, rinsed, and subjected to desorption in 0.1 mol·L^−1^ EDTA for 2 h. The regenerated adsorbent was reused in subsequent cycles under identical conditions. Removal efficiency for each cycle was calculated relative to the first cycle.

#### 2.5.1. Adsorption Kinetics Study

The adsorption kinetics of Cr(VI) onto CS-AC were investigated using pseudo-first-order and pseudo-second-order kinetic models. Kinetic experiments were conducted under the optimal conditions (pH 1.0, adsorbent dosage 2 g·L^−1^, temperature 25 °C, agitation speed 250 rpm) with contact times ranging from 10 to 90 min at various initial Cr(VI) concentrations. The pseudo-first-order (Equation (2)) and pseudo-second-order (Equation (3)) kinetic models are expressed as follows:

(2) $$\ln\left(q_{e}-q_{t}\right)=lnq_{e}-k_{1}t$$

(3) $$\frac{t}{q_{t}}=\frac{1}{K_{2}q_{e}^{2}}+\frac{t}{q_{e}}$$

where *q_t_* (mg·g^−1^ min^−1^) is the amount of Cr adsorbed at time *t* (min), *k*_1_ (min^−1^) and *k*_2_ (g·mg^−1^·min^−1^) are the rate constants of pseudo-first-order and pseudo-second-order equations.

The best-fitting kinetic model was determined by comparing the regression coefficients (R^2^) obtained from the linearized plots of each model.

#### 2.5.2. Adsorption Thermodynamics Study

Adsorption isotherms describe the relationship between the equilibrium concentration of Cr(VI) in solution and the amount adsorbed per unit mass of adsorbent under constant conditions. They provide critical insights into the adsorption mechanism and are essential for evaluating equilibrium data using various theoretical models. In this study, the Langmuir and Freundlich models were applied to fit the experimental equilibrium data. The Langmuir and Freundlich isotherm models are expressed by Equations (4) and (5), respectively:

(4) $$\frac{C_{e}}{q_{e}}=\frac{1}{K_{L}q_{m}}+\frac{C_{e}}{q_{m}}$$

(5) $$lnq_{e}=lnK_{F}+\frac{1}{n}{lnC}_{e}$$

## 3. Results and Discussion

### 3.1. Characteristics of CS-AC Adsorbents

**Figure 1:**
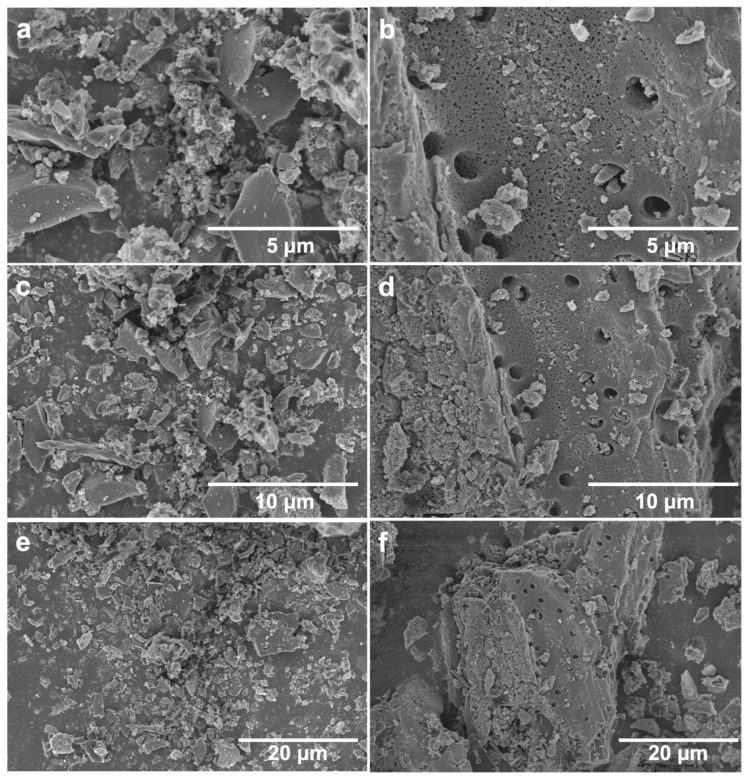
FE-SEM images of coconut shell activated carbon (CAC) before and after chitosan modification: pristine CAC (**a**,**c**,**e**) and chitosan-modified activated carbon (CS-AC) (**b**,**d**,**f**).

The morphologies of CAC and CS-AC were examined by SEM, and the images are shown in Figure 1. The pristine CAC exhibits irregular blocky structures with rough surfaces and loosely distributed particles (Figure 1a,c,e). In contrast, the CS-AC sample displays a smoother and more leveled surface morphology with well-developed porous structures (Figure 1b,d,f). The morphological change is attributed to the removal of surface impurities during alkaline washing, resulting in a smoother morphology for CS-AC. The well-developed pore structure contributes to a larger specific surface area, providing more active sites for the adsorption of Cr(VI). The energy-dispersive X-ray spectroscopy (EDS) mapping was utilized to analyze the nitrogen distribution on the surface of both pristine CAC and CS-AC. As shown in Figure S1a, the nitrogen element distribution on the surface of pristine CAC is extremely sparse, with only a few scattered points detected. In contrast, the EDS mapping of CS-AC (Figure S1b) reveals a dense and uniform distribution of nitrogen across the entire surface region. This homogeneous nitrogen signal confirms the successful loading and even coating of CS onto the activated carbon matrix. The introduction of nitrogen-rich CS provides abundant amino groups (-NH_2_), which serve as active sites for the adsorption of Cr(VI) species through electrostatic attraction, complexation, and reduction mechanisms. These results are consistent with the FTIR analysis and further support the effective functionalization of activated carbon by CS.

**Figure 2:**
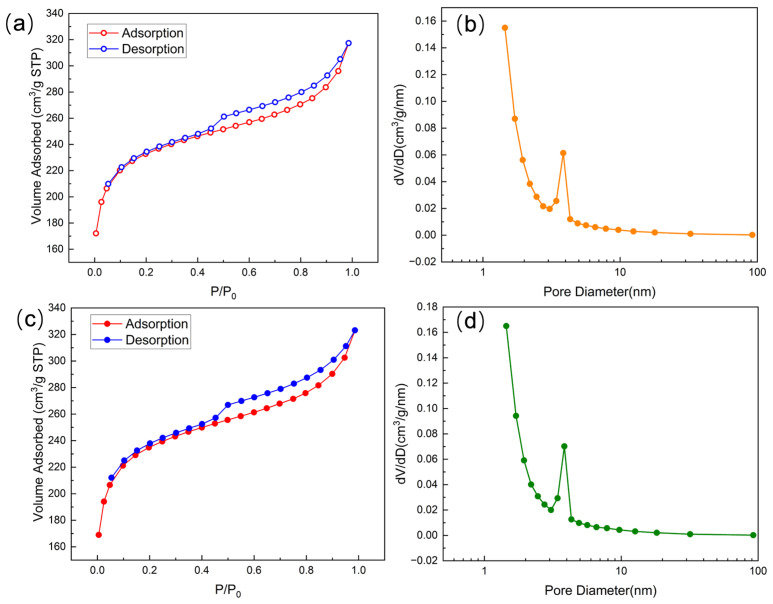
N_2_ adsorption-desorption isotherms and pore-size distribution curves of pristine CAC (**a**,**b**) and CS-AC (**c**,**d**).

The nitrogen adsorption-desorption isotherms and corresponding pore size distribution curves of CAC and CS-AC are shown in Figure 2. As presented in Figure 2a,c, both adsorbents exhibit Type IV isotherms with a noticeable hysteresis loop, indicating the coexistence of microporous and mesoporous structures [29]. This pore structure is favorable for adsorption processes in aqueous systems. For pristine CAC (Figure 2a), a sharp increase in nitrogen uptake at low relative pressures (P/P_0_ < 0.1) suggests the presence of abundant micropores. The gradual increase in adsorption capacity at intermediate and high relative pressures, along with the hysteresis loop, confirms the existence of mesopores, which can serve as transport channels to facilitate the diffusion of adsorbate species toward internal adsorption sites. After chitosan modification, CS-AC (Figure 2c) maintained a similar isotherm shape, indicating that the overall pore framework of the activated carbon was preserved. Moreover, the total nitrogen adsorption capacity of CS-AC is slightly higher than that of pristine CAC across the entire relative pressure range, owing to the alkaline washing step during modification, which effectively removes surface impurities and ash residues and thus creates additional pores.

The pore size distribution curves (Figure 2b,d) further illustrate the structural differences between the two materials. The CS-AC sample exhibits a dominant pore size distribution in the microporous region (<2 nm), along with a fraction of mesopores centered around 2–5 nm. Such a hierarchical pore structure is beneficial for adsorption, as micropores provide a high surface area while mesopores enhance mass transfer. In contrast, CAC shows a lower micropore volume, which is consistent with the nitrogen adsorption results and confirms that chitosan modification leads to a slightly higher specific surface area and total pore volume.

The specific surface area and pore structure characteristics of CS-AC and CAC are summarized in Table 1. Both adsorbents exhibit high BET surface areas and well-developed pore structures, which are typical of coconut shell-derived activated carbons. The pristine CAC shows a BET specific surface area of 743.928 m^2^/g, with a total pore volume (V_tot_) of 0.492 cm^3^/g and a micropore volume (V_mic_) of 0.255 cm^3^/g. The significant micropore contribution is consistent with the sharp nitrogen uptake observed at low relative pressures in the adsorption isotherm, while the average pore size of 2.65 nm indicates a micro-mesoporous structure favorable for adsorption applications. After chitosan modification, the BET surface area of CS-AC increases slightly to 753.559 m^2^/g, accompanied by increases in total pore volume (0.501 cm^3^/g) and micropore volume (0.266 cm^3^/g). This increase is attributable to the alkaline washing step during modification, which effectively removes surface impurities and ash residues and thus opens additional pores. In addition to the enhanced textural properties, chitosan modification introduces abundant amine (-NH_2_) and hydroxyl (-OH) functional groups onto the activated carbon surface. These functional groups serve as effective binding sites for Cr(VI) species through electrostatic attraction, complexation, and hydrogen bonding, particularly under acidic conditions where Cr(VI) predominantly exists as negatively charged oxyanions.

**Figure 3:**
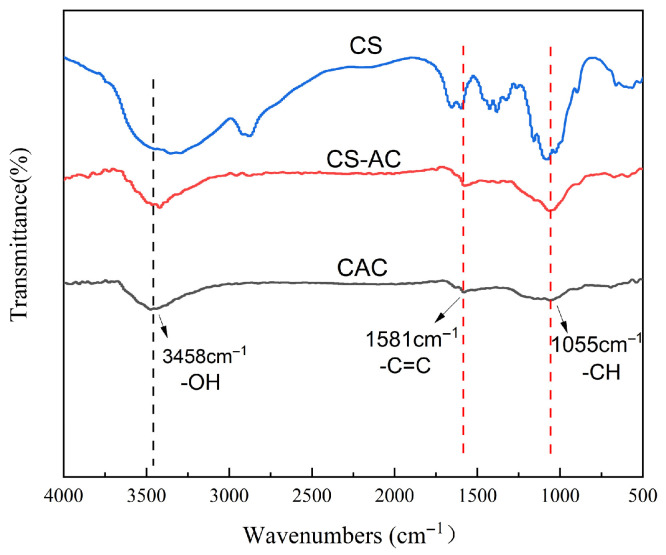
FTIR spectra of pristine CAC, CS, and chitosan-modified activated carbon (CS-AC).

The characteristic functional groups of CS, CAC, and CS-AC were investigated by FTIR, and the spectra are shown in Figure 3. For CS, the broad peak at 3458 cm^−1^ is attributed to the coupled stretching and vibration of N-H and O-H groups. The peak at 1632 cm^−1^ corresponds to the stretching vibration of C=O in -COOH groups [30]. The bending vibration of C-H appears around 1384 cm^−1^, while the peaks at 1055 cm^−1^ and 887 cm^−1^ are assigned to the stretching vibration of C-O and the characteristic peak of -COC-, respectively [31]. Compared to CAC, CS-AC exhibits peaks at 1055 cm^−1^ and 887 cm^−1^, confirming that chitosan was successfully loaded onto the activated carbon. FTIR analysis thus confirms the presence of abundant amino and hydroxyl groups on the CS-AC surface, which can interact with Cr(VI) ions through surface complexation or coordination bonding, contributing to chemisorption. The dominance of the pseudo-second-order kinetic model further supports the involvement of chemical interactions in the adsorption process.

### 3.2. Adsorption Behavior of Cr(VI)

**Figure 4:**
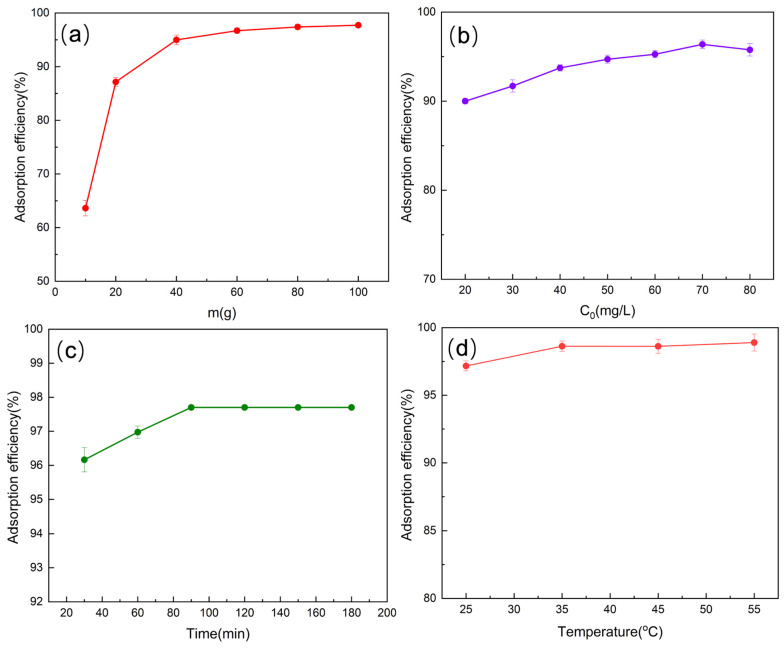
Effect of adsorbent dosage (**a**), initial Cr(VI) concentration (**b**), time (**c**), and temperature (**d**) on the removal efficiency of Cr(VI) by CS-AC.

The effects of adsorbent dosage, initial Cr(VI) concentration, contact time, and temperature on the adsorption performance of CS-AC are presented in Figure 4. As shown in Figure 4a, the removal efficiency of Cr(VI) increased markedly with increasing adsorbent dosage, reaching an optimum at 80 mg, which is attributed to the greater availability of active adsorption sites and functional groups. Beyond this point, the removal efficiency plateaued, indicating that the adsorption process became limited by the available Cr(VI) concentration rather than the number of adsorption sites. Figure 4b illustrates that an increase in the initial Cr(VI) concentration led to higher adsorption capacity but a slight decrease in removal efficiency, which can be ascribed to the saturation of active sites and intensified competition among Cr(VI) ions at higher concentrations. As shown in Figure 4c, adsorption was rapid during the initial stage, followed by a gradual approach to equilibrium at approximately 90 min. This behavior suggests that surface adsorption dominated the early stage, while intraparticle diffusion and site saturation governed the later stage. Regarding the effect of temperature (Figure 4d), the removal efficiency of Cr(VI) slightly increased as the temperature rose from 25 °C to 55 °C. Higher temperatures enhance the mobility of Cr(VI) ions in solution, facilitating mass transfer and increasing the probability of interaction with active adsorption sites [32]. Additionally, elevated temperature may promote the activation of surface functional groups, thereby strengthening adsorption interactions [33]. Nevertheless, the relatively small variation in removal efficiency indicates that CS-AC can effectively remove Cr(VI) even at ambient temperature, which is advantageous for practical applications.

**Figure 5:**
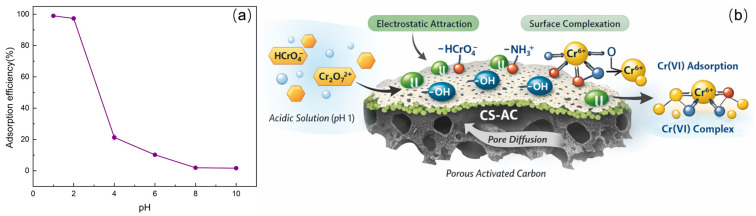
Effect of solution pH on the adsorption of Cr(VI) by CS-AC (**a**), and the adsorption mechanism of Cr(VI) on CS-AC (**b**).

The solution pH plays a crucial role in the adsorption behavior of Cr(VI) on CS-AC, as illustrated in Figure 5a. The removal efficiency reaches a maximum value of 99.04% at pH 1.0 and then decreases sharply with increasing pH, indicating a strong pH-dependent adsorption process. This phenomenon is closely related to both the speciation of Cr(VI) in aqueous solution and the surface charge characteristics of CS-AC. Under strongly acidic conditions (pH ≤ 2), Cr(VI) predominantly exists as negatively charged oxyanions such as HCrO_4_^−^ and Cr_2_O_7_^2−^. Meanwhile, the amino groups introduced onto the CS-AC surface via chitosan modification are readily protonated (-NH_2_ → -NH_3_^+^), resulting in a positively charged adsorbent surface. To evaluate the effectiveness of chitosan modification, the Cr(VI) adsorption performance of pristine CAC was compared with that of CS-AC under identical conditions. As shown in Figure S2, the pristine CAC exhibited a removal efficiency of only 76.26% at pH 1.0. In contrast, the CS-AC composite achieved a significantly higher removal efficiency of 99.05% under the same conditions. Meanwhile, the BET surface area of CS-AC (753.56 m^2^/g) is only slightly higher than that of pristine CAC (743.93 m^2^/g), as shown in Table 1. This suggests that the introduction of chitosan-derived functional groups plays a dominant role in the adsorption process. The kinetic analysis further supports this point, as the adsorption of Cr(VI) onto CS-AC follows the pseudo-second-order model (R^2^ > 0.99), indicating that chemisorption-mediated by the abundant amino (-NH_2_) and hydroxyl (-OH) groups on the CS-AC surface-is the rate-controlling step, rather than physical diffusion processes governed solely by surface area. Therefore, the substantial enhancement in adsorption capacity is predominantly attributed to the chemical functionalization imparted by chitosan, rather than the modest increase in specific surface area.

As schematically illustrated in Figure 5b, this charge difference gives rise to strong electrostatic attraction between CS-AC and Cr(VI) species, significantly enhancing the adsorption efficiency. In addition to electrostatic interactions, surface complexation also contributes to Cr(VI) adsorption. The protonated amino groups (-NH_3_^+^) and hydroxyl groups (-OH) on CS-AC can interact with Cr(VI) oxyanions through coordination bonding and hydrogen bonding, leading to the formation of stable surface complexes. Furthermore, the well-developed porous structure of CS-AC facilitates pore diffusion, allowing Cr(VI) species to access internal active sites, thereby further improving adsorption performance under acidic conditions. Beyond electrostatic attraction and surface complexation, the reduction of Cr(VI) to Cr(III) is another plausible mechanism contributing to the overall removal process, as has been suggested in the literature [34]. Several indirect observations support the occurrence of partial reduction. First, the maximum adsorption efficiency was achieved under strongly acidic conditions (pH 1–2), where the amino groups (-NH_2_) of chitosan are fully protonated to -NH_3_^+^. Under such conditions, these protonated amine groups can act as electron donors, facilitating the reduction of Cr(VI) to the less toxic Cr(III) [35]. Second, the adsorption kinetics were well described by the pseudo-second-order model, indicating that chemisorption, potentially involving electron transfer and reduction reactions, is the rate-controlling step [36]. Similar adsorption–reduction mechanisms have been extensively documented for chitosan-modified materials, where the hydroxyl and amino groups synergistically contribute to both the capture and detoxification of Cr(VI) [37]. Therefore, the overall removal mechanism of Cr(VI) by CS-AC is proposed to involve a combination of electrostatic attraction, surface complexation, and possible partial reduction inferred from indirect evidence (pH dependence, kinetics, FTIR changes). Conclusive confirmation of the redox process would require further investigations, such as analysis of the chromium valence states on the adsorbent surface.

### 3.3. Adsorption Isotherms and Kinetic Analysis

#### 3.3.1. Adsorption Kinetics

**Figure 6:**
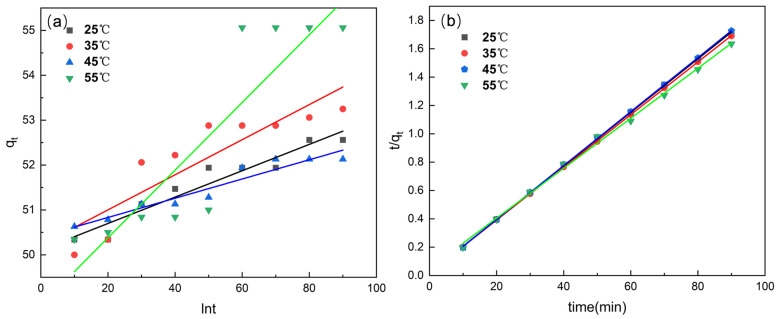
Pseudo-first-order (**a**) and pseudo-second-order (**b**) kinetic model fitting for Cr(VI) adsorption onto CS-AC at different temperatures and times.

The adsorption kinetics of Cr(VI) onto CS-AC were evaluated using pseudo-first-order and pseudo-second-order kinetic models. As shown in Figure 6, the pseudo-second-order model provides an excellent fit to the experimental data, with correlation coefficients (R^2^) exceeding 0.99 at all investigated temperatures. In contrast, the pseudo-first-order model shows relatively lower R^2^ values and poorer agreement between the calculated and experimental adsorption capacities. These results suggest that the adsorption of Cr(VI) onto CS-AC is predominantly governed by chemisorption involving valence forces through electron sharing or exchange between Cr(VI) species and surface functional groups. Similar kinetic behavior has been widely reported for chitosan-based adsorbents used for heavy metal removal. It should be noted that linearization of kinetic models may introduce bias by transforming the original data, which can affect error distribution and lead to less accurate parameter estimation. In particular, the pseudo-second-order model often appears to provide a better fit under linearized conditions. Nevertheless, linearized models are still widely used in the literature due to their simplicity and ease of parameter determination, and they provide a useful basis for preliminary evaluation and comparison of adsorption behavior.

#### 3.3.2. Adsorption Isotherms

**Figure 7:**
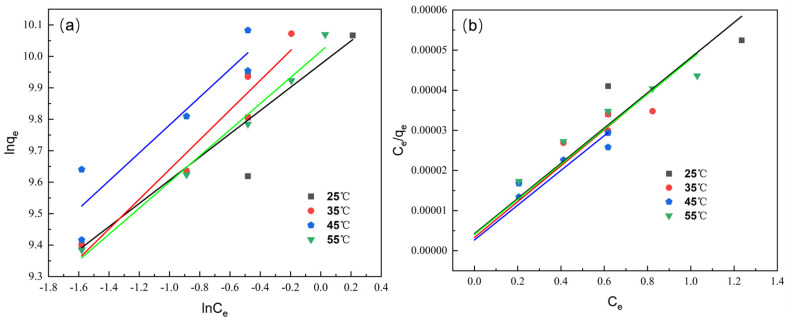
Freundlich (**a**) and Langmuir (**b**) adsorption isotherm fitting for Cr(VI) adsorption onto CS-AC at different temperatures.

The equilibrium adsorption behavior of Cr(VI) onto CS-AC was analyzed using the Langmuir and Freundlich isotherm models. As shown in Figure 7, the Langmuir model exhibits a better correlation with experimental data compared to the Freundlich model, as indicated by higher R^2^ values. The maximum monolayer adsorption capacity (q_m_) derived from the Langmuir model ranges from approximately 220 to 230 mg·g^−1^, which is comparable to or even higher than previously reported values for chitosan-modified carbonaceous adsorbents. As summarized in Table S1, chitosan-modified biochar exhibited a maximum adsorption capacity of 197 mg·g^−1^ at pH 3 [38], while Fe/CSCC activated carbon showed a capacity ranging from 200 to 240 mg·g^−1^ [39], chitosan–clay composite bead (17.31 mg·g^−1^) and Terminalia catappa-derived activated carbon (TCAC) (184.45 mg·g^−1^) [40,41], and activated carbons derived from waste leaves of *Rhus typhina* and *Amorpha fruticosa* exhibited maximum Langmuir adsorption capacities for Cr(VI) of 266.54 mg·g^−1^ under similar acidic conditions [42]. In contrast, C-ZLCH achieved a relatively lower capacity of 105 mg·g^−1^ at pH 6.7 [43]. The CS-AC composite developed in this work demonstrates a competitive adsorption capacity of 220–230 mg·g^−1^ under strongly acidic conditions (pH 1), highlighting its potential as an efficient adsorbent for Cr(VI) removal from acidic industrial wastewater. Beyond adsorption capacity, the performance of CS-AC under strongly acidic conditions (pH 1–2) is notable. Many chitosan-based adsorbents reported in the literature are optimized at pH 3–6 [38,39,43], whereas CS-AC achieves 99.05% removal at pH 1.0, demonstrating its suitability for highly acidic industrial effluents. In terms of reusability, most modified chitosan adsorbents retain 73–95% of their initial capacity after 3–5 cycles [44,45]; CS-AC retains 98.10% after five cycles, which is at the higher end of the reported range. Regarding structural stability, crosslinked chitosan systems generally exhibit superior acid resistance [46], yet our non-crosslinked CS-AC preserved its characteristic FTIR peaks after five cycles, indicating that the coating remained largely intact under the tested conditions despite the absence of chemical crosslinkers. These comparisons position CS-AC as a simple, low-cost, yet effective alternative for Cr(VI) removal under strongly acidic conditions. The novelty of this work lies not solely in achieving a high adsorption capacity, but in demonstrating that a simple, non-crosslinked chitosan modification on coconut shell activated carbon yields excellent Cr(VI) removal performance (99.05% efficiency) under strongly acidic conditions (pH 1–2). This result indicates that the adsorption of Cr(VI) onto CS-AC occurs via monolayer coverage on energetically homogeneous adsorption sites. The Freundlich model, which assumes heterogeneous surface adsorption, shows relatively lower fitting accuracy, suggesting that surface heterogeneity plays a less dominant role in the adsorption process. Overall, the Langmuir isotherm model provides a more appropriate description of Cr(VI) adsorption behavior on CS-AC.

As detailed in Section 2.4, the selective DPC method was used to measure only Cr(VI). Therefore, all reported removal efficiencies and adsorption capacities refer specifically to Cr(VI), and total chromium was not measured. The possible reduction of Cr(VI) to Cr(III) is inferred from several indirect experimental observations. It is important to emphasize that while these observations are consistent with a reduction process, they do not constitute direct proof. The maximum adsorption efficiency occurred under strongly acidic conditions (pH 1–2), where the amino groups of chitosan are protonated and can act as electron donors. The adsorption kinetics followed the pseudo-second-order model, indicating a chemisorption process that may involve electron transfer. FTIR analysis showed shifts and intensity decreases in the -NH_2_/-OH bands, along with the appearance of a new band around 890 cm^−1^ after Cr(VI) uptake, suggesting chemical interactions such as coordination and redox reactions.

**Figure 8:**
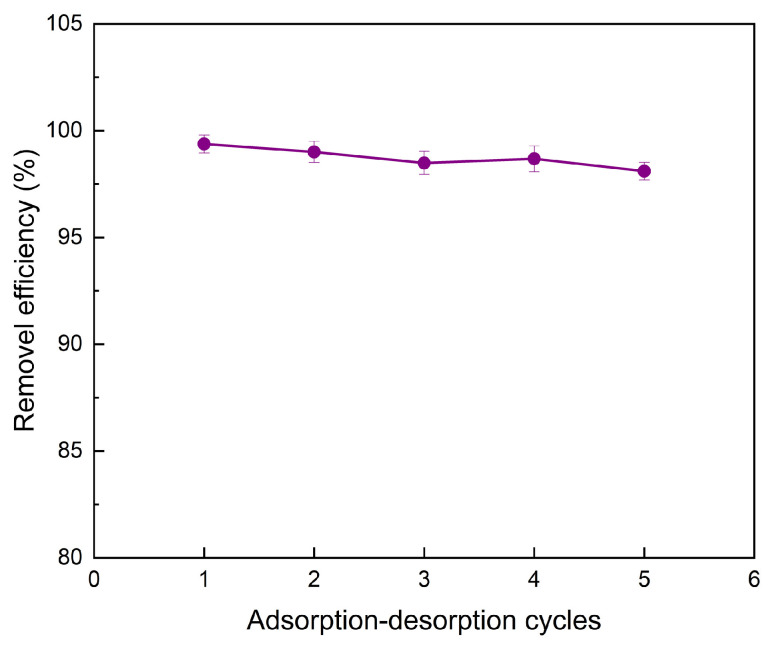
Adsorption-desorption experiment of Cr(VI) by CS-CA.

To further evaluate the reusability of CS-AC, adsorption–desorption experiments were conducted, and the results are presented in Figure 8. The CS-AC composite exhibited excellent regeneration performance, retaining a high adsorption efficiency of 98.10% after five consecutive cycles of EDTA-assisted desorption, indicating good stability and reusability for Cr(VI) removal. The FTIR spectra of CS-AC before and after Cr(VI) adsorption are presented in Figure S3. After adsorption of Cr(VI), several changes were observed in the FTIR spectrum. The peak at 3450 cm^−1^ shifted slightly to a higher wavenumber and decreased in intensity, suggesting the involvement of -OH and -NH_2_ groups in the adsorption process. The intensity of the amide I band (1650 cm^−1^) diminished, which may indicate that part of the amino groups participated in the reduction of Cr(VI) to Cr(III) [47]. Moreover, a new weak band appeared around 890 cm^−1^, which can be assigned to the formation of Cr-N or Cr-O bonds, providing direct evidence for surface complexation and partial reduction of Cr(VI). These spectral changes collectively suggest that the adsorption of Cr(VI) onto CS-AC involves not only electrostatic attraction but also chemical interactions such as coordination and redox reactions [48]. Importantly, the retention of the characteristic FTIR peaks of chitosan after five cycles (Figure S3) correlates well with the preserved adsorption efficiency (98.10%), suggesting that the key functional groups responsible for Cr(VI) capture remain largely active after repeated use. Furthermore, the presence of background electrolytes may influence mass transfer processes and surface charge characteristics of the adsorbent [49]. These effects could alter adsorption kinetics and equilibrium behavior, highlighting the need for systematic investigation under realistic environmental conditions.

To evaluate the stability of the chitosan coating under the strongly acidic conditions employed (pH 1–2), EDS mapping was performed on CS-AC before and after five consecutive adsorption–desorption cycles. As shown in Figure S4, the surface of CS-AC exhibited a uniform distribution of nitrogen with an elemental content of 7.39% before cycling, derived exclusively from the chitosan coating since the pristine CAC contains no intrinsic nitrogen. After five cycles of Cr(VI) adsorption and EDTA-assisted desorption, the nitrogen content remained largely unchanged at 6.84%, with only a slight decrease of 0.55%. The results suggested that the chitosan coating appeared to remain largely intact under the experimental conditions employed. It should be acknowledged that chitosan is known to be soluble in acidic media, and crosslinking is generally required to maintain its structural integrity at pH < 2. In the present study, CS-AC was prepared without chemical crosslinkers, and the experiments were conducted at room temperature. While the EDS and FTIR results indicate no substantial loss of nitrogen or functional groups after five cycles, the possibility of partial dissolution or gradual leaching over extended cycling cannot be completely excluded. Future work employing crosslinking strategies or direct leaching measurements would further validate the long-term stability of the coating.

## 4. Conclusions

A chitosan-modified coconut shell activated carbon (CS-AC) was successfully synthesized and demonstrated exceptional efficiency for Cr(VI) removal from water. Under optimized conditions, the composite achieved a removal efficiency of 99.04%, with adsorption data fitting the pseudo-second-order kinetic and Langmuir isotherm models, indicating a chemisorption-driven monolayer process. Its high reusability (98.10% after five cycles), combined with its low cost and environmental compatibility, highlights the significant potential of CS-AC as a sustainable adsorbent for Cr(VI) removal. Nevertheless, it should be noted that chitosan can be partially soluble under strongly acidic conditions (pH 1–2) in the absence of crosslinking. Although our EDS and FTIR analyses suggest that the coating remained largely intact under the tested conditions, the material’s long-term stability under continuous operation would benefit from further validation, such as leaching tests or crosslinking strategies. Furthermore, this study was conducted using synthetic solutions; additional validation using real industrial wastewater is necessary to confirm practical applicability.

## Figures and Tables

**Table 1:** Comparison of specific surface area and average pore diameter before and after modification.

Sample	S_BET_/ (m^2^/g)	V_tot_/ (cm^3^/g)	V_mic_/ (cm^3^/g)	Average Pore Size/(nm)
CS-AC	753.559	0.501132	0.266073	2.66008
CAC	743.928	0.492145	0.255003	2.6462

## Data Availability

The original contributions presented in this study are included in the article/Supplementary Materials. Further inquiries can be directed to the corresponding authors.

## Supplementary Materials

The following supporting information can be downloaded at: https://www.mdpi.com/article/10.3390/polym18101237/s1, Figure S1: EDS nitrogen element mapping of (a) pristine CAC and (b) CS-AC; Figure S2: Effect of solution pH on the adsorption efficiency of Cr(VI) by CS-AC and pristine CAC. Adsorption conditions: initial Cr(VI) concentration 70 mg·L^−1^, adsorbent dosage 80 mg, contact time 90 min, temperature 25 °C; Figure S3: FTIR spectra of CS-AC before and after 5 cycles Cr(VI) adsorption; Figure S4: EDS nitrogen element mapping of CS-AC before (a) and after (b) 5 cycles Cr(VI) adsorption; and Table S1: Comparison of adsorption performance of various adsorbents for Cr(VI) removal. References [38,39,40,41,42,43] are cited in the Supplementary Materials.
