# Supplementary material for: Chitosan-Modified Coconut Shell Activated Carbon for Efficient Hexavalent Chromium Removal from Aqueous Solution
Source: Polymers (Basel). 2026 May 19;18(10):1237. doi: 10.3390/polym18101237 (PMC13210409; doi:10.3390/polym18101237)
Supplement: Supplementary file 1 [file polymers-18-01237-s001.zip › polymers-4183918-supplementary.pdf]

Supporting Information

Chitosan-Modified Coconut Shell Activated Carbon for Efficient Hexavalent Chromium Removal from Aqueous Solution

Danyun Lei[a*], Weiyi She[a], Xiaoyu Chen[a], Lei You[a], Ying Zheng[a], Byoung-Suhk Kim [b,c**]

[a] College of Urban Construction, Wuchang Shouyi University, Wuhan 430064, PR China

[b] Department of Organic Materials & Textile Engineering, Jeonbuk National University, Jeonju-si, Jeollabuk-do 561-756, Republic of Korea

[c] Department of BIN Convergence Technology, Jeonbuk National University, Jeonju-si, Jeollabuk-do 561-756, Republic of Korea

* Correspondence: 2020111017@wsyu.edu.cn (D. Lei)

** Correspondence: kbsuhk@jbnu.ac.kr (B. S. Kim)

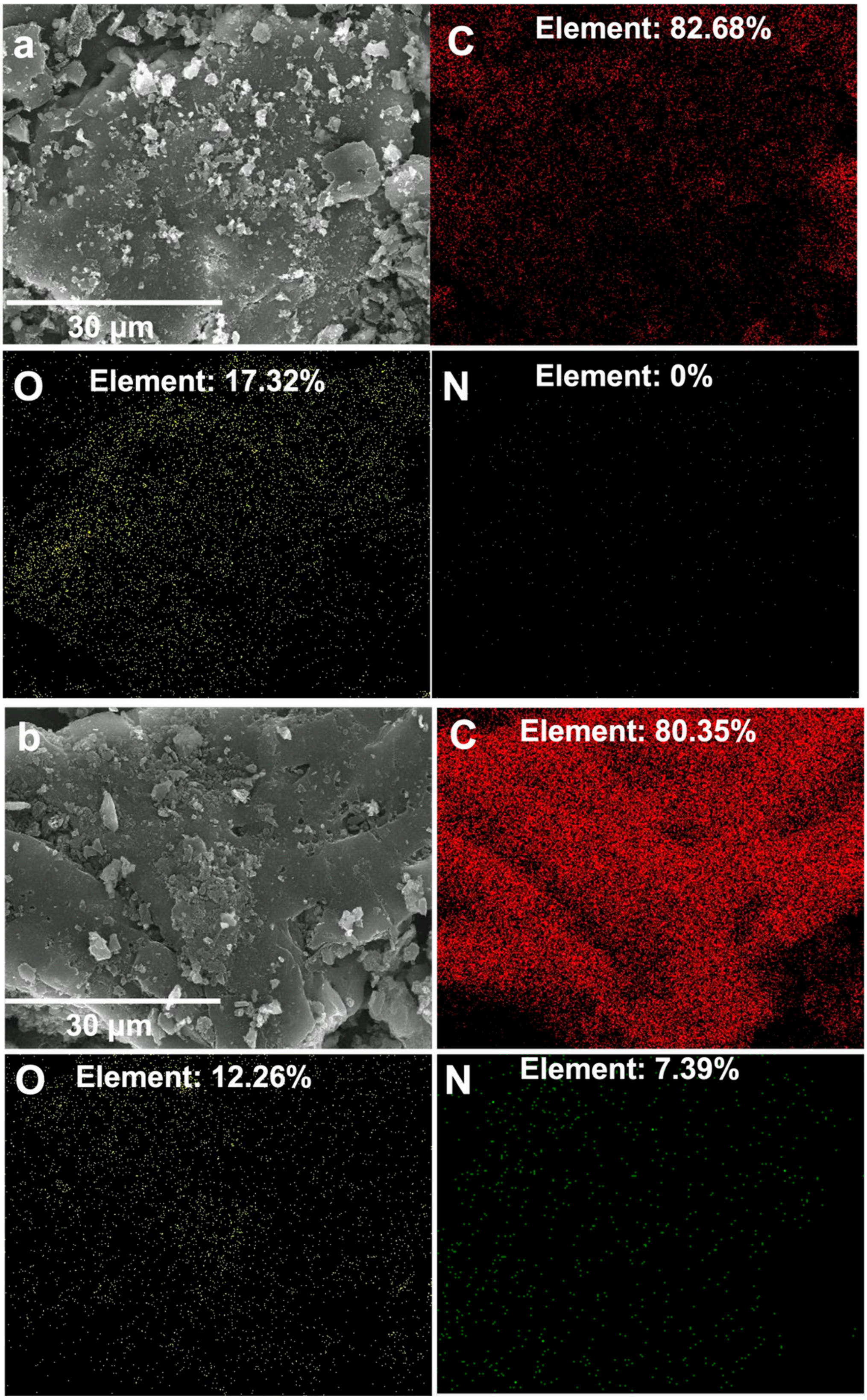

Figure S1. EDS nitrogen element mapping of (a) pristine CAC and (b) CS-AC.

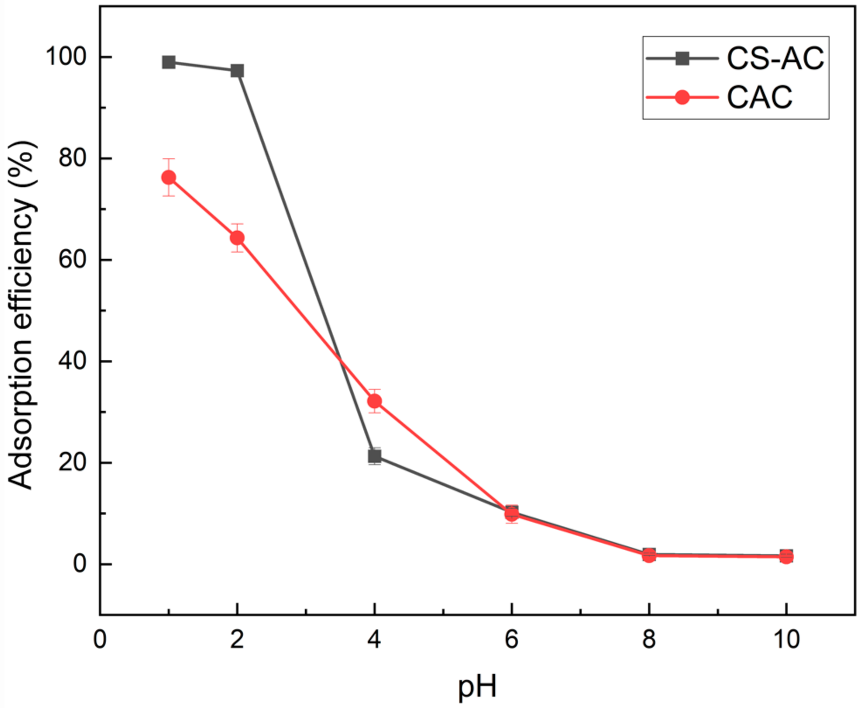

Figure S2. Effect of solution pH on the adsorption efficiency of Cr(VI) by CS-AC and pristine CAC. Adsorption conditions: initial Cr(VI) concentration 70 mg·L^{-1}, adsorbent dosage 80 mg, contact time 90 min, temperature 25 °C.

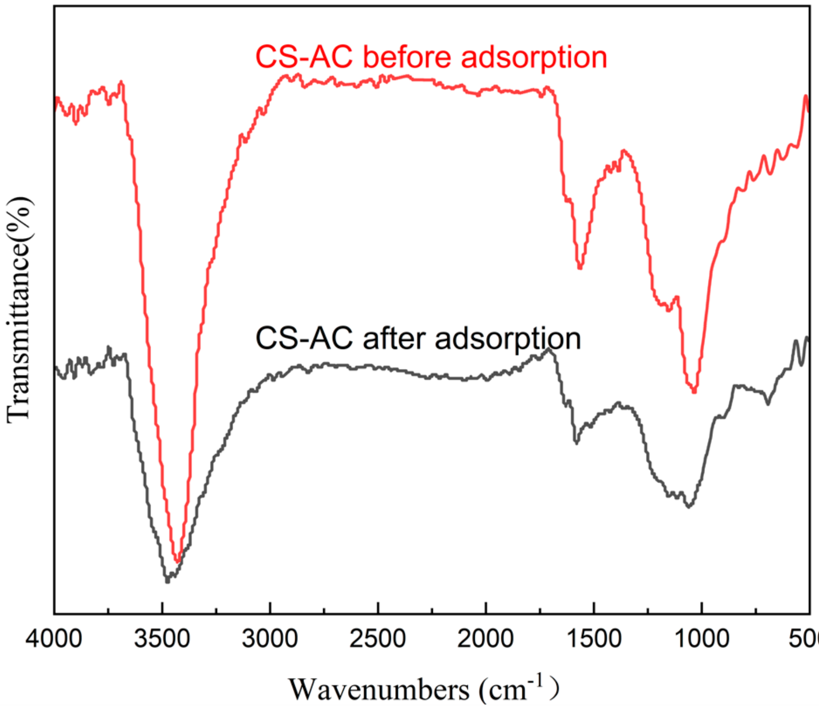

Figure S3. FTIR spectra of CS-AC before and after 5 cycles Cr(VI) adsorption.

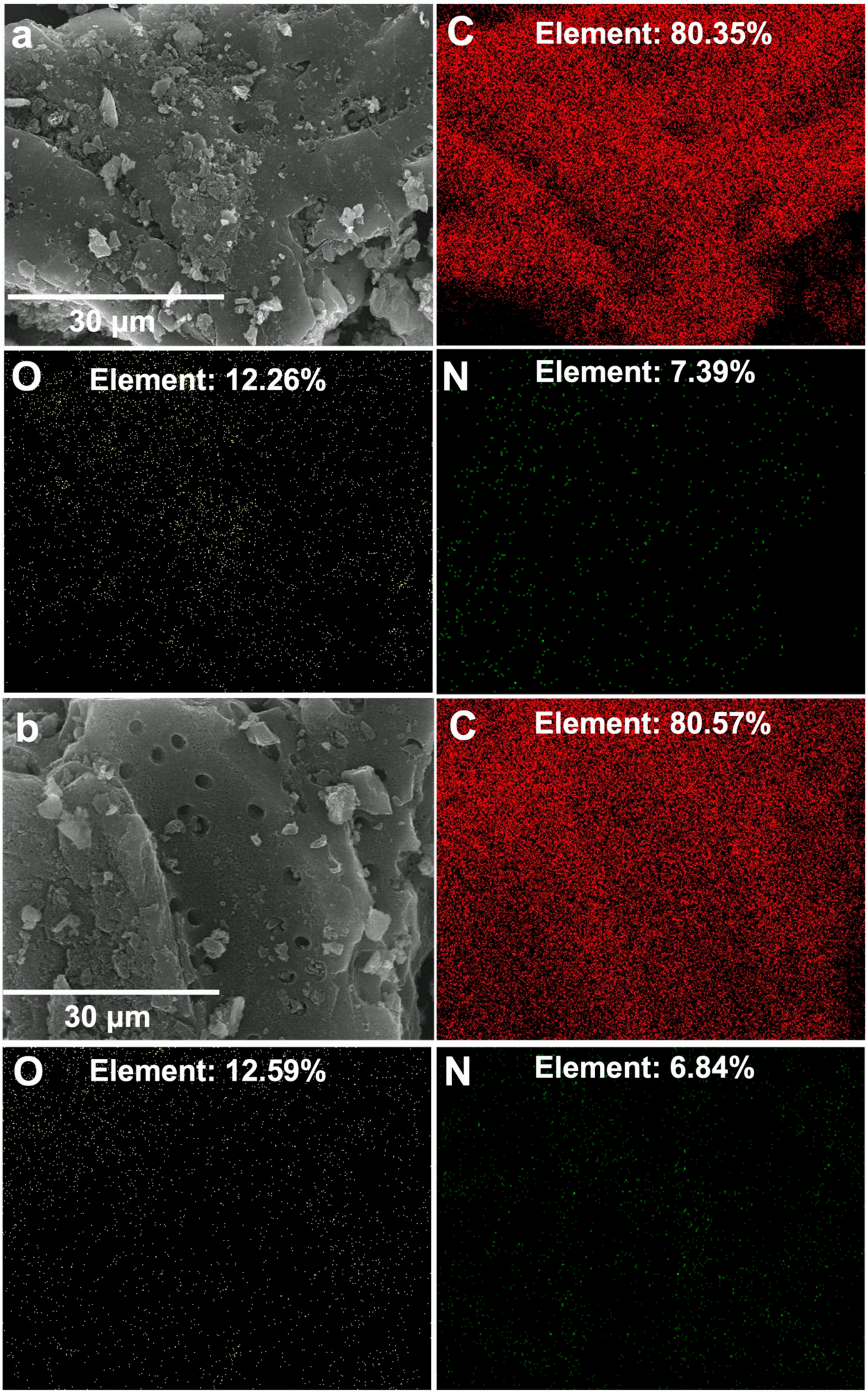
a
30 μm
C
Element: 80.35%
O
Element: 12.26%
N
Element: 7.39%
b
30 μm
C
Element: 80.57%
O
Element: 12.59%
N
Element: 6.84%

Figure S4. EDS nitrogen element mapping of CS-AC before (a) and after (b) 5 cycles Cr(VI) adsorption.

Table S1. Comparison of adsorption performance of various adsorbents for Cr(VI) removal.

Adsorbent	q_{max}(mg/g)	Conditions	References
Chitosan-modified biochar	197	pH3	[38]
Fe/CSCC	200-240	pH3	[39]
chitosan–clay composite bead	17.31	pH3	[40]
TCAC	184.45	pH3	[41]
ACRT1.0	266	pH2	[42]
C-ZLCH	105	pH6.7	[43]
CS-AC	220-230	pH1	This work

References [38-43] are cited in the main text.
